# Association between TGF-β1 Polymorphisms and Head and Neck Cancer Risk: A Meta-Analysis

**DOI:** 10.3389/fgene.2017.00169

**Published:** 2017-11-03

**Authors:** Quan Shi, Xing Wang, Chuan Cai, Shuo Yang, Na Huo, Hongchen Liu

**Affiliations:** ^1^Institute of Stomatology, Chinese PLA General Hospital, Beijing, China; ^2^Stomatological Hospital, Shanxi Medical University, Taiyuan, China

**Keywords:** head and neck cancer, transforming growth factor beta 1, single nucleotide polymorphisms, risk factor, meta-analysis

## Abstract

**Background and Objective:** Studies have been conducted to explore the association between the single nucleotide polymorphisms (SNPs) in transforming growth factor beta 1 (TGF-β1) and head and neck cancer (HNC) susceptibility, however the findings are still inconclusive. Therefore, we conduct this meta-analysis to quantitatively assess the association.

**Methods:** Embase and PubMed were searched for all eligible clinical studies. The odds ratio (OR) and 95% confidence interval (CI) of each study were pooled to estimate the association between SNPs in the TGF-β1 and the HNC risk. Subgroup analysis was used to explore whether particular characteristics were related to the value of overall ORs and 95% CIs.

**Results:** Seven case-control studies, including three SNPs (−509C/T, 869T/C, and 915G/C), were examined. Overall, this meta-analysis failed to identify a significant association between TGF-β1−509C/T, 915G/C polymorphism and HNC risk in any models. As for the 869T/C polymorphism, significant associations were observed in the allelic model (C vs. T: OR = 1.351, 95%CI: 1.030–1.772), the homozygote model (CC vs. TT: OR = 1.585, 95%CI: 1.026–2.449) and the dominant model (CT/CC vs. TT: OR = 1.398, 95%CI: 1.008–1.937). This polymorphism was also found in the Asian group as well (C vs. T: OR = 1.400, 95%CI: 1.003–1.956, CC vs. TT: OR = 1.814, 95%CI: 1.018–3.233).

**Conclusion:** Meta-analysis failed to show a statistical association between TGF-β1−509C/T, 915G/C polymorphism, and HNC risk in any genetic models. However, it was found that TGF-β1 869C/T polymorphism may be involved in susceptibility to HNC, especially in Asian patients. However, given the limitations of this meta-analysis, further well-designed studies are required in the future.

## Introduction

Head and neck cancer (HNC) is a type of cancer within the mouth, nose, sinuses, salivary glands, throat, and lymph nodes in the neck. As the sixth most common cancer and ninth most frequent cause of cancer-related death, HNC affected more than 4.6 million people worldwide in 2013, and it has a high morbidity and a low survival rate (Petersen, 2009; Brunotto et al., 2014; Chai et al., 2015; Global Burden of Disease Study 2013 Collaborators, 2015). Incidence of HNC is increasing worldwide and according to the World Health Organization (WHO) this trend is expected to continue into the next several decades (Bettendorf et al., 2004). HNC occurs most often in men in their 50s or 60s, but in recent years the incidence among younger individuals has increased (Bray et al., 2008). It has been shown that smoking, alcohol consumption, viral infection with Epstein-Barr virus (EBV) or human papillomavirus (HPV), and environmental exposure are the primary etiologic factors contributing to HNC. Likewise, genetic susceptibility also plays a critical role in the development of HNC (Brunotto et al., 2014; Lacko et al., 2014; Munshi et al., 2015).

As the most common form of genetic variation in humans, single nucleotide polymorphism (SNP) in genes encoding for susceptibility factors may influence gene expression, protein function, and disease predisposition (Nachman, 2001; Hsu et al., 2014). Many studies have shown that SNPs in some genes may contribute to an individual's susceptibility to cancer, including HNC. Therefore, identification of SNPs, which serve as genetic susceptibility markers of tumors, has become a recent research interest. According to available evidence, SNPs in biotransformation enzymes (Shukla et al., 2012), DNA repair genes (Li et al., 2016), apoptotic pathways (Ma et al., 2011), alcohol metabolism (Bediaga et al., 2015), and immune inflammatory cytokines (Singh et al., 2015; Ma and Zhou, 2016) could affect the risk of HNC. Furthermore, these SNPs may also play a role in prognostication and may serve as a predictive tool in making treatment decisions for HNC (Zafereo et al., 2009; Lundberg et al., 2012; Sivadas et al., 2013; Zou et al., 2014).

Transforming growth factor beta 1 (TGF-β1) is a member of the transforming growth factor beta superfamily of cytokines. TGF-β1 can regulate both the immune system and cellular functions, including cell differentiation, cell proliferation, extracellular matrix production, apoptosis, and angiogenesis (Massagué, 2012). TGF-β1 also plays an important role in the carcinogenesis of various tumors. Genetic variations in the promoter region of the TGF-β1 gene may have an effect on transcription and protein synthesis. At present,−509C/T and 869T/C are the most commonly studied polymorphisms in TGF-β1. Recent studies have shown that SNPs of TGF-β1 are associated with susceptibility to a large range of cancers, including lung cancer (Fan et al., 2014), prostate cancer (Cai et al., 2014), gastric cancer (Chang et al., 2014), and hepatocellular cancer (Lu et al., 2016). Clinical studies have also been conducted to explore the association between SNPs in TGF-β1 and HNC susceptibility (Hu et al., 2012; Carneiro et al., 2013; Hsu et al., 2015; Khaali et al., 2016). However, findings are still inconclusive. Some results are even contradictory, particularly when the subjects are of the same ethnicity.

Considering that a single study, due to the small number of subjects, may be insufficient to provide a reliable conclusion, we performed a meta-analysis of all eligible studies in the hope of obtaining a more precise estimation of the association between the SNPs in TGF-β1 and a correlative HNC risk. The results of this meta-analysis may provide clinicians with better evidence-based evaluations and give patients guidance for early preventive care.

## Materials and methods

### Literature search

A comprehensive computer literature search was performed to identify studies on the association between gene polymorphisms of TGF-β1 and the risk of HNC. Embase and PubMed were searched on November 11, 2016 for eligible studies, with no restrictions on publication languages and dates. A combination of the following key words and Mesh terms was used: head and neck cancer; nasopharyngeal cancer; oral cancer; oropharyngeal cancer; laryngeal cancer; transforming growth factor; TGF; polymorphism. Additional studies were identified by a hand search of references of related studies and reviews.

### Inclusion and exclusion criteria

The studies must conform to the following criteria to be eligible: (1) they should be clinical studies focusing on the associations between TGF-β1 gene polymorphism and the risk of head and neck cancers; (2) the studies should provide sufficient data, including frequencies of alleles or genotypes in case and control groups to estimate the odds ratio (OR) value and 95% confidence interval (CI); (3) the cancer patients and control subjects are described and confirmed clearly in the studies; (4) the studies should use validated genotyping methods. The exclusion criteria were: (1) reviews or case reports, animal studies or introductory studies; (2) clinical studies not focused on the associations between TGF-β1 gene polymorphism and the risk of head and neck cancers; (3) studies with no available data reported, and duplicated reports.

According to the inclusion and exclusion criteria above, two reviewers (QS and XW) independently assessed potentially eligible studies. At first, irrelevant records were excluded after the titles and abstracts were examined, and then full-texts of potential interest were scanned. Any disagreement was resolved by discussing with a third reviewer (NH).

### Data extraction

The following basic data were collected from the studies by two reviewers (QS and XW) independently: the first author, year of publication, design type of the study, ethnicity (Asian, Caucasian or other population), the type of cancer, description of study population (sample size, age, and sex), genotyping method, SNPs in each study, alleles or genotypes frequency, and the results of Hardy–Weinberg equilibrium (HWE).

### Methodological assessment

To evaluate the quality of the included studies, a methodological quality assessment scale adjusted from previous publications (Camargo et al., 2006; Li et al., 2014) was adopted and assessed by two reviewers independently (CC and SY; Table S1). In the assessment scale, representativeness of cases, source of controls, sample size, quality control of genotyping methods, adjusted factors, and HWE results in the control subjects were used to appraise the methodological quality of the included studies. The scores of this scale range from 0 to 10, with 0 to 4, 5 to 7, and 8 to 10 indicating poor, moderate, and good study quality, respectively.

### Statistical analyses

Statistical analyses were performed using STATA software (Version 12.0; Stata Corp, College Station, Texas, USA). Pooled OR-value and 95%CI were calculated to evaluate the association between SNPs: −509C/T (rs18800469), 869T/C (rs1982073), and 915G/C (rs1800471) in TGF-β1 and HNC risk. Pooled ORs were performed for five models: allelic model (−509C/T: T vs. C; 869T/C: C vs. T; 915 G/C: C vs. G); homozygote model (−509C/T: TT vs. CC; 869T/C: CC vs. TT; 915 G/C: CC vs. GG); heterozygote model (−509C/T: TC vs. CC; 869T/C: CT vs. TT; 915 G/C: CG vs. GG); dominant model (−509C/T: TC/TT vs. CC; 869T/C: CT/CC vs. TT; 915 G/C: CG/CC vs. GG); and recessive model (−509C/T: TT vs. TC/CC; 869T/C: CC vs. CT/TT; 915 G/C: CC vs. CG/GG). Subgroup analyses were performed to explore whether particular characteristics in the studies (ethnicity, HWE, quality score) were related to the overall ORs and 95% CIs if there was a sufficient number of studies. HWE in each study was tested by Chi-square test. A *P*-value below 0.05 was considered statistically significant.

The statistical heterogeneity was verified by *I*^2^ statistics. If the *I*^2^-value was <50%, it would suggest that the heterogeneity was low and a fixed-effect model was adopted to estimate the OR and 95%CI, otherwise, the random effects was used. Sensitivity analysis was made by removing one study each time in order to analyze the stability of the pooled results, if there were enough studies.

## Results

### Study selection and characteristics

**Figure 1:**
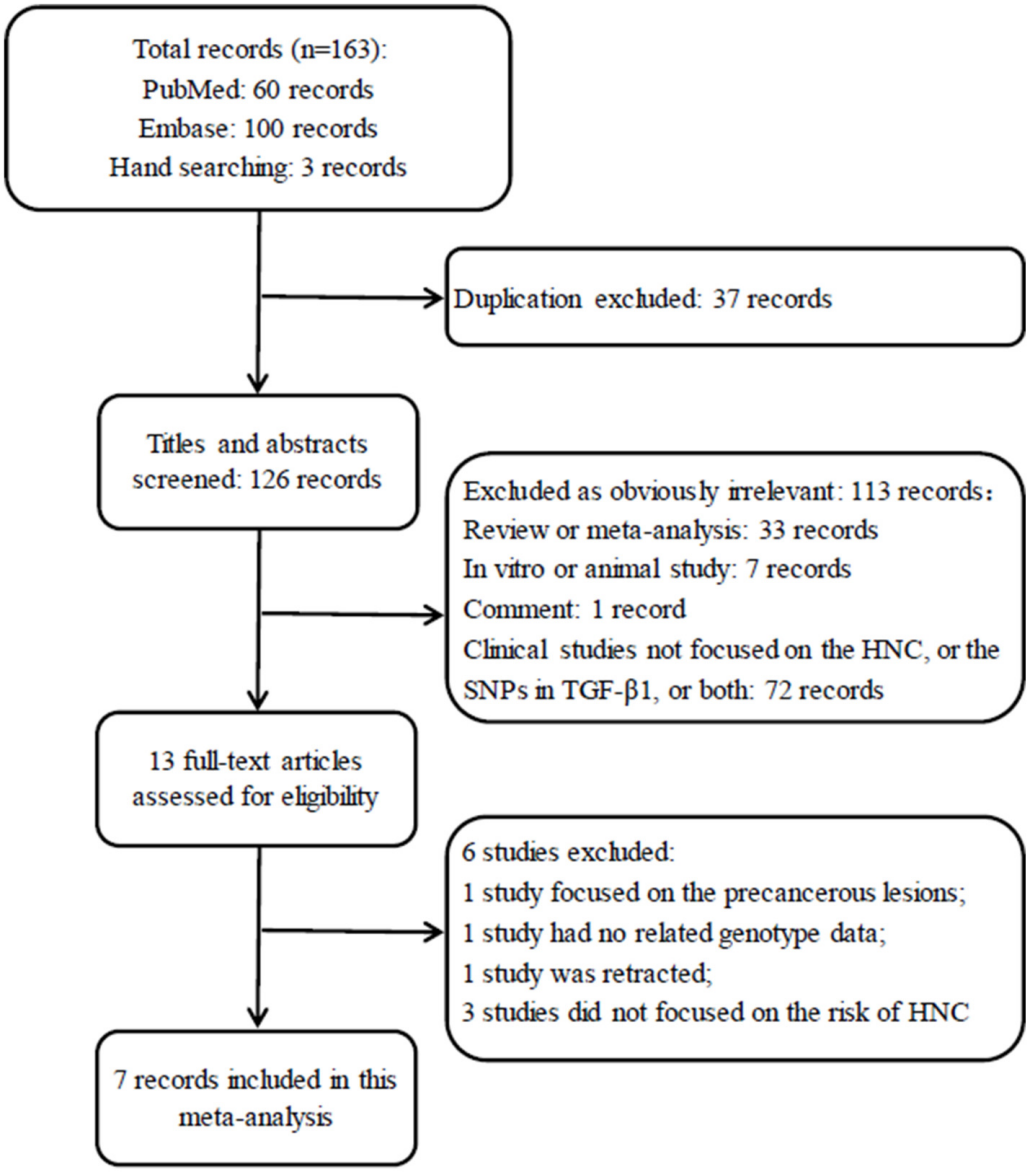
Flow diagram for selection of studies. HNC, head and neck cancers.

A total of 163 records were identified after an initial search using Embase, PubMed and hand searching. After excluding duplicated records, 126 studies were left for screening. Of those, 113 articles obviously irrelevant to SNP in TGF-β1 gene and HNC risk were excluded after the titles and abstracts were read, leaving 13 articles for a further full-text review. Finally, 7 studies (Wei et al., 2007; Gaur et al., 2011; Al-Hadyan et al., 2012; Hu et al., 2012; Carneiro et al., 2013; Hsu et al., 2015; Khaali et al., 2016) were identified for this meta-analysis according to the inclusion criteria. The flow diagram of search processes and results of included studies are shown in Figure 1.

All 7 included studies were case-control studies, and their publication dates ranged from 2007 to 2016. In total, 1534 cancer patients and 1744 control subjects were studied. Three studies (Wei et al., 2007; Hu et al., 2012; Khaali et al., 2016) reported on TGF-β1−509C/T polymorphism. Two of them focused on Asians populations (Wei et al., 2007; Hu et al., 2012), while the third one focused on North Africans (Khaali et al., 2016). Six studies (Wei et al., 2007; Gaur et al., 2011; Al-Hadyan et al., 2012; Hu et al., 2012; Carneiro et al., 2013; Khaali et al., 2016) reported on TGF-β1 869T/C polymorphism, four of which involved Asian subjects (Wei et al., 2007; Gaur et al., 2011; Al-Hadyan et al., 2012; Hu et al., 2012) and the other two involved Brazilians (Carneiro et al., 2013) and North Africans (Khaali et al., 2016). Two studies (Gaur et al., 2011; Hsu et al., 2015) reported on TGF-β1 915G/C polymorphism and both of them were conducted among Asians. The distribution of genotypes for the three SNPs in the controls of all studies was consistent with HWE, except for one study reporting on TGF-β1 869T/C (Carneiro et al., 2013). Six included studies (Wei et al., 2007; Gaur et al., 2011; Al-Hadyan et al., 2012; Hu et al., 2012; Hsu et al., 2015; Khaali et al., 2016) had a quality score ≥5 (moderate-high quality), while one study (Carneiro et al., 2013) had poor quality (the study deprived from HWE). All of the studies have reported that the study has been approved by the related committees. The general characteristics of the studies included in this meta-analysis are summarized in Table 1.

**Table 1:** Characteristics of studies included in the meta-analysis.

**First author**	**Year**	**Country**	**Type of cancer**	**Case**		**Control**		**Genotyping method**	**Adjusted factors**	**SNP**	**HWE (*P*-value)**	**Quality**
				**Number** **(Male/Female)**	**Age**	**Number** **(Male/Female)**	**Age**					
Khaali W	2016	Morocco	NPC	384 (263/121)	42.24^a^	361 (243/118)	43.58^a^	PCR-RFLP	Sex, age household in childhood	−509 C/T 869 T/C	0.92 0.61	8
Hsu HJ	2015	China	OC	162 (125/37)	52.70 ± 12.38	128 (77/51)	59.20 ± 12.91	PCR-SSP	Age, smoking, drinking, betel quid chewing	915 G/C	0.18	7
Carneiro NK	2013	Brazil	OC	62 (62/0)	57.26 ± 11.30	62 (62/0)	43.44 ± 11.09	PCR	—	869 T/C	0.01	4
Hu S	2012	China	NPC	522 (315/207)	46^b^	712 (404/308)	47^b^	PCR-RFLP	Age, sex	−509 C/T 869 T/C	0.39 0.99	7
Al-Hadyan KS	2012	Saudi Arabia	Mixed	156 (122/34)	50^b^	251 (191/60)	47^b^	PCR	—	869 T/C	0.92	5
Gaur P	2011	India	OC	140 (119/21)	51.4 ± 13.6	120 (NR)	NR	PCR-RFLP	Age, sex, ethnicity	869 T/C 915 G/C	0.29 0.20	8
Wei YS	2007	China	NPC	108 (76/32)	49.8 ± 10.5	120 (82/38)	48.3 ± 11.2	PCR-RFLP	Age, sex, smoking status	−509 C/T 869 T/C	1 0.98	7

*SNP, single nucleotide polymorphism; HWE, Hardy–Weinberg equilibrium; NPC, nasopharyngeal carcinoma; OC, oral cancer; PCR-RFLP, polymerase chain reaction restriction fragment length polymorphism; PCR-SSP, polymerase chain reaction sequence-specific primer; NR, not report*.

a *The mean age of the subjects*.

b *The median age of the subjects*.

### Meta-analysis results

#### −509C/T

**Figure 2:**
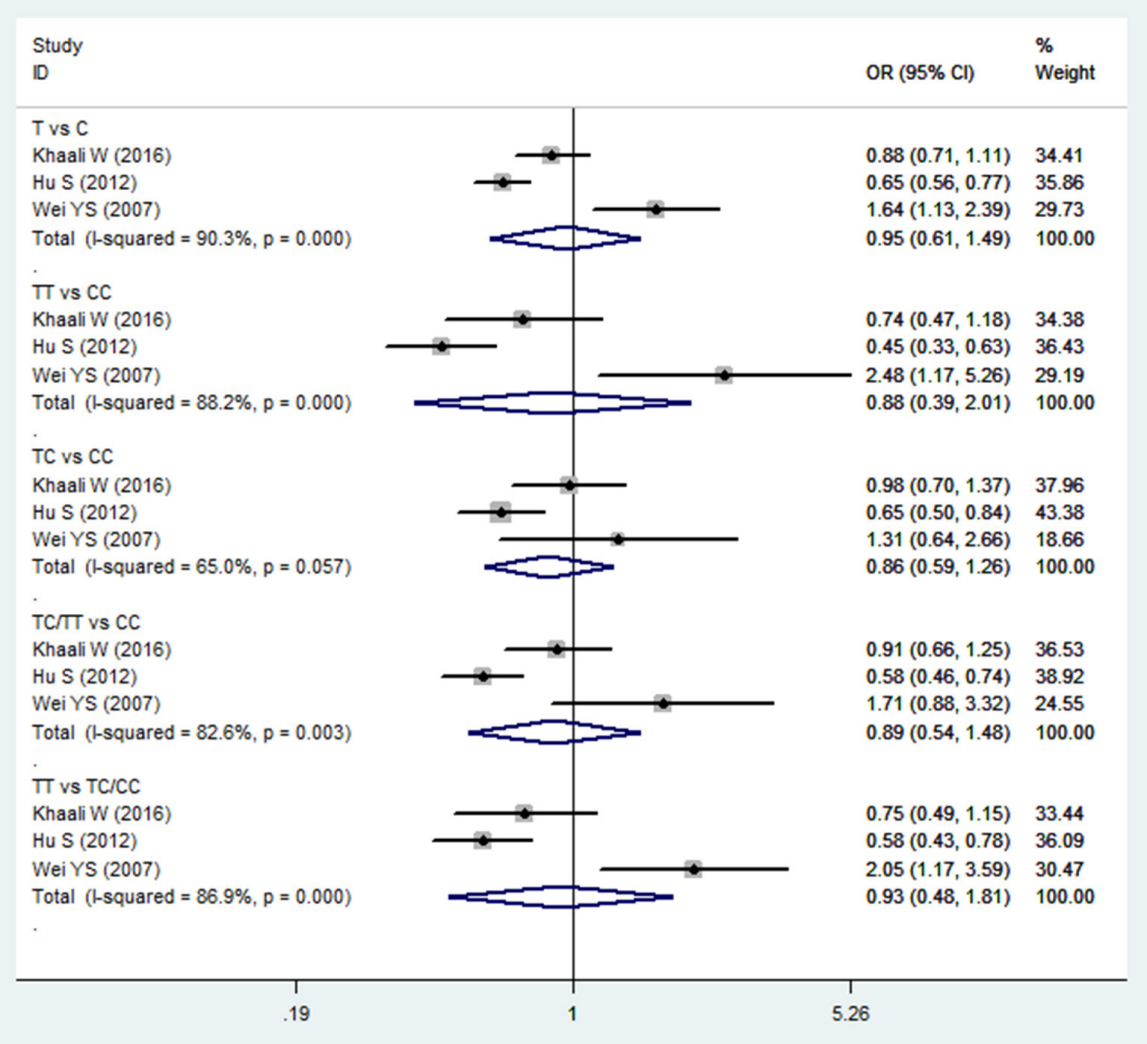
Forest plot of the TGF-β1−509C/T polymorphism and HNC risk in all comparison models. OR, odds ratio, CI, confidence interval.

The meta-analysis of TGF-β1−509C/T polymorphism was based on 3 studies (Wei et al., 2007; Hu et al., 2012; Khaali et al., 2016). Because the statistical heterogeneity between them was high (all *I*^2^-values were more than 50%), the random effects model was applied. Overall, this meta-analysis failed to identify a significant association between TGF-β1−509C/T polymorphism and HNC risk in terms of both allele frequency and genotype distribution (T vs. C: OR = 0.954, 95%CI: 0.612–1.488, *P* = 0.837; TT vs. CC: OR = 0.882, 95%CI: 0.388–2.007, *P* = 0.765; TC vs. CC: OR = 0.864, 95%CI: 0.591–1.262, *P* = 0.449; TC/TT vs. CC: OR = 0.894, 95%CI: 0.539–1.483, *P* = 0.663; TT vs. TC/CC: OR = 0.930, 95%CI: 0.478–1.811, *P* = 0.832; Figure 2 and Table 2). Furthermore, subgroup analysis based on ethnicity showed no significant association in TGF-β1−509C/T polymorphism and HNC risk between Asian groups and other populations.

**Table 2:** Statistics for polled ORs and subgroup analysis.

**Subgroup**		**OR** **(95%CI)**	***P*-value**	***I*^2^** **(%)**	**OR** **(95%CI)**	***P*-value**	***I*^2^** **(%)**	**OR** **(95%CI)**	***P*-value**	***I*^2^** **(%)**	**OR** **(95%CI)**	***P*-value**	***I*^2^** **(%)**	**OR** **(95%CI)**	***P*-value**	***I*^2^** **(%)**
−**509 C/T**		**T vs. C**			**TT vs. CC**			**TC vs. CC**			**TC/TT vs. CC**			**TT vs. TC/CC**		
Total	3	0.954 (0.612–1.488)	0.837	90.3	0.882 (0.388–2.007)	0.765	88.2	0.864 (0.591–1.262)	0.449	65.0	0.894 (0.539–1.483)	0.663	82.6	0.930 (0.478–1.811)	0.832	86.9
Asians	2	1.021 (0.414–2.516)	0.964	94.9	1.023 (0.194–5.392)	0.978	93.9	0.849 (0.435–1.656)	0.631	69.7	0.952 (0.333–2.716)	0.926	88.7	1.067 (0.310–3.665)	0.918	93.5
Others ^a^	1	0.884 (0.707–1.105)	0.279	–	0.742 (0.465–1.184)	0.211	–	0.977 (0.698–1.368)	0.891	–	0.912 (0.664–1.253)	0.571	–	0.752 (0.490–1.155)	0.193	–
**869 T/C**		**C vs. T**			**CC vs. TT**			**CT vs. TT**			**CT/CC vs. TT**			**CC vs. CT/TT**		
Total	6	**1.351** **(1.030–1.772)**	**0.030**	80.1	**1.585** **(1.026–2.449)**	**0.038**	71.8	1.279 (0.957–1.709)	0.097	56.0	**1.398** **(1.008–1.937)**	**0.044**	69.9	1.314 (0.953–1.810)	0.095	62.5
Asians	4	**1.400** **(1.003–1.956)**	**0.048**	83.1	**1.814** **(1.018–3.233)**	**0.043**	75.6	1.184 (0.963–1.455)	0.115	49.4	1.435 (0.953–2.159)	0.083	71.3	1.588 (1.000–2.428)	0.050	70.5
Others^a^	2	1.48 (0.52–4.19)	0.46	83.4	1.282 (0.498–3.299)	0.606	71.4	1.643 (0.532–5.073)	0.388	81.5	1.502 (0.542–4.166)	0.434	81.5	0.916 (0.653–1.285)	0.611	0
HWE^b^	5	1.273 (0.976–1.660)	0.075	80.8	1.514 (0.947–2.420)	0.083	75.2	1.129 (0.948–1.345)	0.175	39.8	1.290 (0.938–1.774)	0.117	68.7	1.350 (0.937–1.946)	0.107	70.0
**915 G/C**		**C vs. G**			**CC vs. GG**			**CG vs. GG**			**CG/CC vs. GG**			**CC vs. CG/GG**		
Total	2	3.094 (0.594–16.119)	0.180	94.5	1.584 (0.799–3.141)	0.188	0	4.15 (0.470–36.682)	0.200	95.3	4.021 (0.544–29.733)	0.173	95.0	1.327 (0.699–2.517)	0.387	5.5

*N, Number of studies; OR, Odds ratio; CI, Confidence interval; P, P-values for pooled ORs; HWE, Hardy–Weinberg equilibrium*.

a *Other ethnicity*.

b *The studies in which Hardy–Weinberg equilibrium in control subjects*.

*The bold values indicate the results of the analysis in these models are of statistical significant*.

#### 869T/C

**Figure 3:**
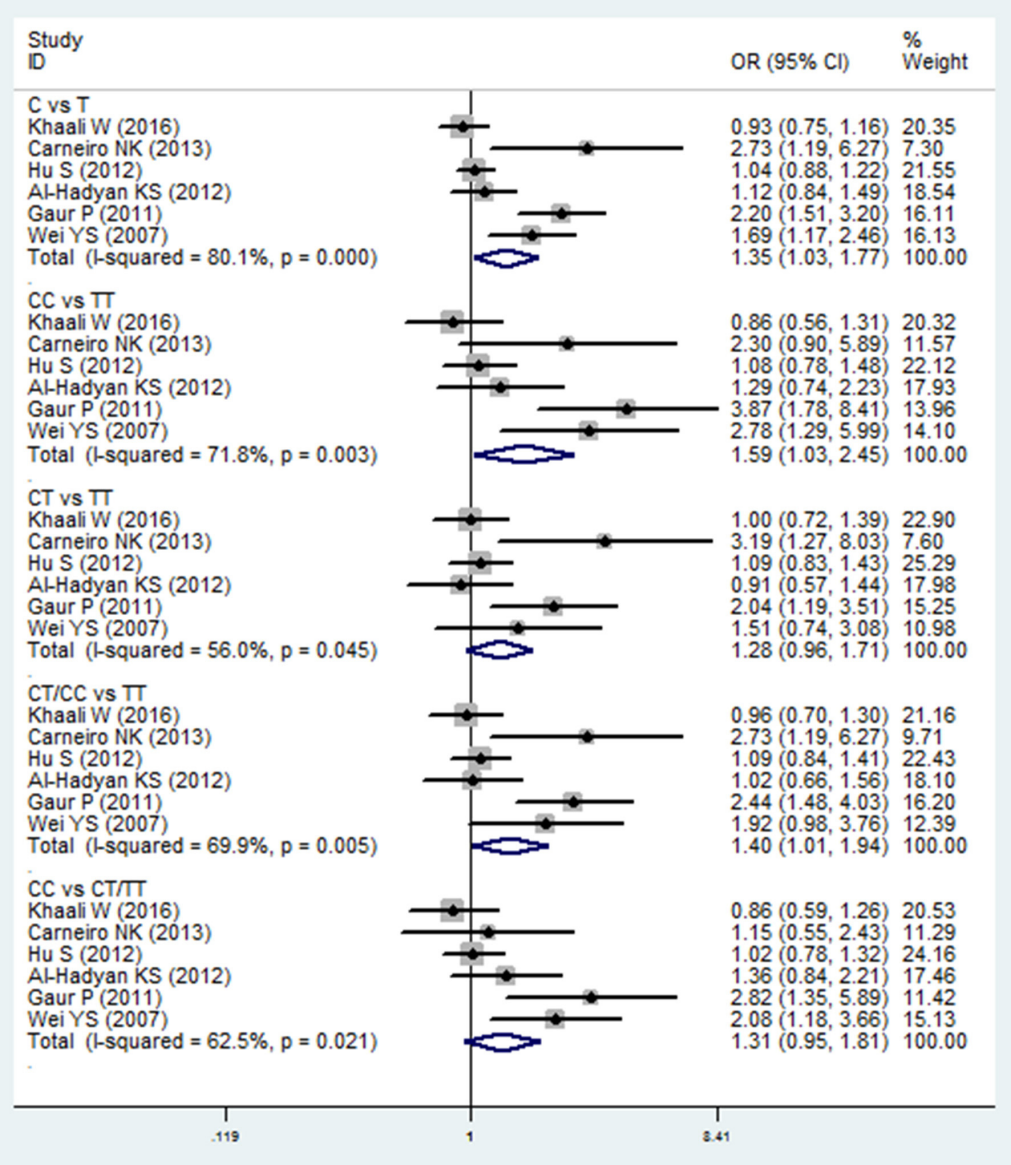
Forest plot of the TGF-β1 869T/C and HNC risk in all comparison models. OR, odds ratio, CI, confidence interval.

Meta-analysis of the ORs between TGF-β1 869C/T polymorphism and HNC risk was performed in six included studies (Wei et al., 2007; Gaur et al., 2011; Al-Hadyan et al., 2012; Hu et al., 2012; Carneiro et al., 2013; Khaali et al., 2016). Significant associations were observed in the allelic model (C vs. T: OR = 1.351, 95%CI: 1.030–1.772, *P* = 0.030), homozygote model (CC vs. TT: OR = 1.585, 95%CI: 1.026–2.449, *P* = 0.038), and dominant model (CT/CC vs. TT: OR = 1.398, 95%CI: 1.008–1.937, *P* = 0.044), as shown in Figure 3 and Table 2. However, no significant association was found in the heterozygote model (CT vs. TT: OR = 1.279, 95%CI: 0.957–1.709, *P* = 0.097) and recessive model (CC vs. CT/TT: OR = 1.314, 95%CI: 0.953–1.810, *P* = 0.095). The random effects model was applied in the comparisons due to high statistical heterogeneity. In this SNP, sensitivity analysis was performed by sequentially removing one study each time. As shown in the Table S2, the results of the pooled OR were robust only in the CT vs. TT model, but were not stable in the comparison of the four models: C vs. T, CC vs. TT, CT/CC vs. TT, and CC vs. CT/TT.

In subgroup analysis, a significant association was observed in the Asian group using the allelic model (C vs. T: OR = 1.400, 95%CI: 1.003–1.956, *P* = 0.048) and the homozygote model (CC vs. TT: OR = 1.814, 95%CI: 1.018–3.233, *P* = 0.043), while there was no significant association in other populations. After removing the study derived from HWE (also the low-quality study; Carneiro et al., 2013), no significant association was found in the overall comparison. The results of subgroup analyses are shown in Table 2.

#### 915G/C

**Figure 4:**
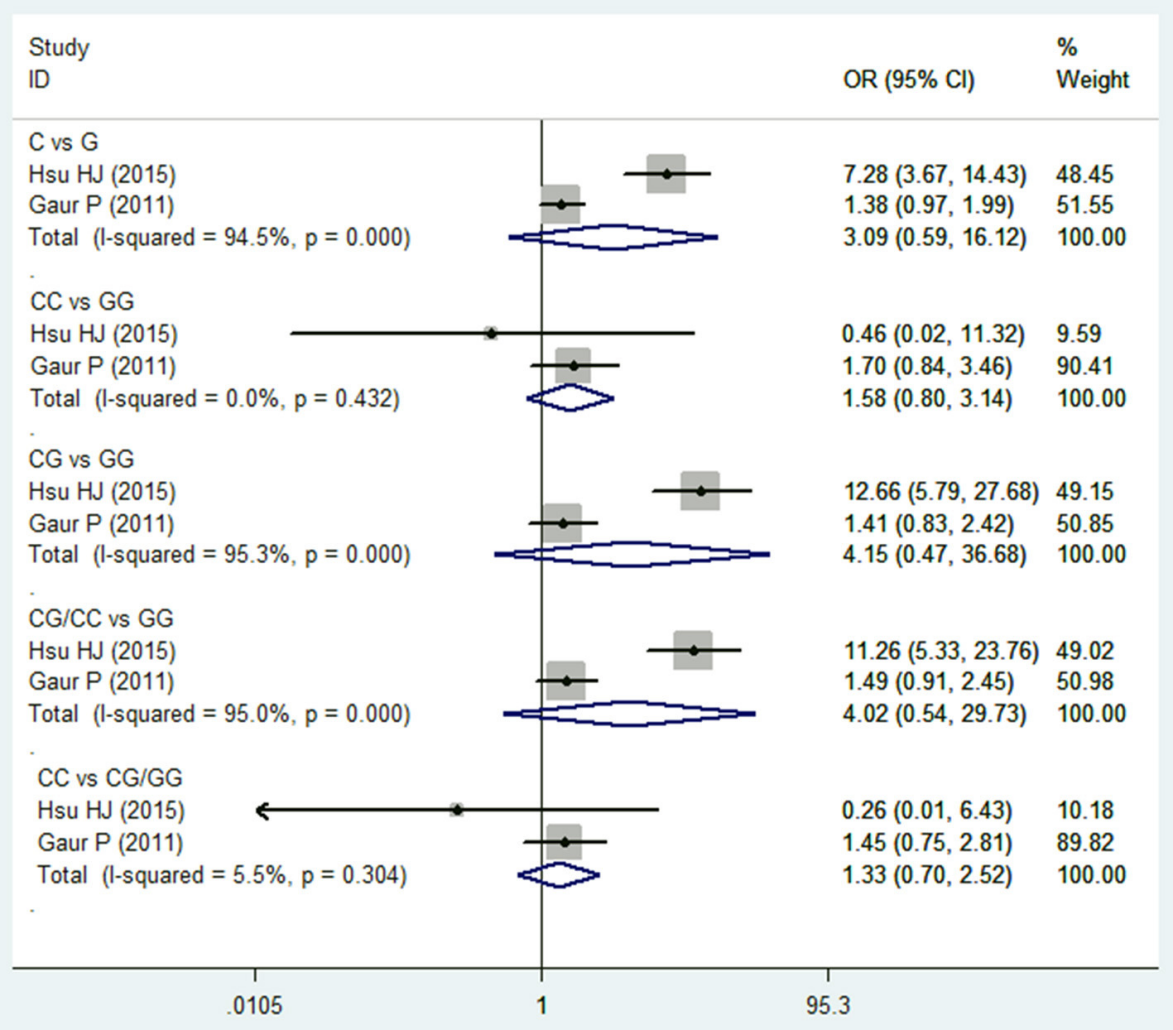
Forest plot of the TGF-β1 915G/C and HNC risk in all comparison models. OR, odds ratio, CI, confidence interval.

Two studies (Wei et al., 2007; Hsu et al., 2015) reported on TGF-β1 915G/C polymorphism and HNC risk. No significant association was observed in the overall comparison (C vs. G: OR = 3.094, 95%CI: 0.594–16.119, *P* = 0.180; CC vs. GG: OR = 1.584, 95%CI: 0.799–3.141, *P* = 0.188; CG vs. GG: OR = 4.15, 95%CI: 0.470–36.682, *P* = 0.200; CG/CC vs. GG: OR = 4.021, 95%CI: 0.544–29.733, *P* = 0.173; CC vs. CG/GG: OR = 1.327, 95%CI: 0.699–2.517, *P* = 0.387; Figure 4 and Table 2). The fixed effect model was applied in the homozygote model and recessive model because the *I*^2^-values were <50%. However, no subgroup analysis was performed due to the limited number of studies.

## Discussion

Inflammation, and expression of relevant cytokines, are important in the promotion, occurrence, and development of HNC. As a multifunctional cytokine, TGF-β1 plays a biphasic role in carcinogenesis. In the early stages, TGF-β1 acts as a cancer inhibitor by regulating epithelial cell proliferation, differentiation, and apoptosis (Rich et al., 2001; Siegel and Massagué, 2003). However, in the advanced and late stages, it acts as a cancer promoter by promoting epithelial to mesenchymal transition, enhancing metalloproteases expression, and increasing cancer motility and angiogenesis (Siegel and Massagué, 2003; Tang et al., 2003). In addition, TGF-β1 may contribute to the aggressive behavior of cancers through the local and systemic immunosuppression effect (Tang et al., 2003). Therefore, the SNPs in TGF-β1 will alter the level of protein expression, which may affect the susceptibility to some diseases, including HNC.

It has been reported that elevated levels of TGF-β1 mRNA and functional protein were identified in the stromal compartment of HNC both *in vitro* and *in vivo* compared with normal tissues, suggesting that TGF-β1 overexpression may provide an HNC promoting microenvironment (Lu et al., 2004; Rosenthal et al., 2004). Though there are studies showing that the C allele in −509C/T polymorphism could increase the level of TGF-β1 expression in serum and in nasopharyngeal carcinoma (NPC) cell lines (Hu et al., 2012), our meta-analysis failed to identify a significant association between TGF-β1−509C/T polymorphism and HNC risk in all of the alleles or genotype models. The same results were also found in the subgroup analysis stratified by ethnicity. The pooled ORs and 95%CIs indicated that TGF-β1−509C/T polymorphism may not affect susceptibility to HNC, but this result should be treated with some caution due to the limited number of included studies focusing on this SNP and high statistical heterogeneity. The conclusions of these three studies are not consistent. Khaali et al. found that in the North African sample, the TGF-β1−509C/T polymorphisms did not substantially influence HNC susceptibility (Khaali et al., 2016). However, the conclusions of the other two studies on Asian samples were the opposite: one concluded the−509T allele carriers were associated with a significantly reduced risk of HNC (Hu et al., 2012), while the other suggested the T allele increased the HNC risk (Wei et al., 2007). Previous studies on the TGF-β1−509C/T polymorphism and risk of cancers were also inconsistent. It has been reported that there was no significant association between the TGF-β1−509C/T polymorphism and risk of gastric cancer (Niu et al., 2012) and breast cancer (Lu et al., 2004). However, based on a meta-analysis of 55 case-control studies, Liu et al. suggested that TGF-β1−509C/T polymorphism might contribute to a decreased risk of colorectal cancer susceptibility, while no association with other cancer risk was identified (Liu et al., 2012). Therefore, the influence of this SNP on cancers may vary from cancer to cancer.

Small but statistically significant associations were observed in the allelic model (C vs. T: OR = 1.351, 95%CI: 1.030–1.772, *P* = 0.030), homozygote model (CC vs. TT: OR = 1.585, 95%CI: 1.026–2.449, *P* = 0.038), and dominant model (CT/CC vs. TT: OR = 1.398, 95%CI: 1.008–1.937, *P* = 0.044) of TGF-β1 869C/T polymorphism. These data indicate that the C allele and the CC genotype may increase the risk of HNC. This may be associated with an increased TGF-β1 level in carriers of the C allele, which might lead to a slightly attenuated immune function and further increase of risk in developing HNC. These findings were similar to those of the two previous meta-analyses. One meta-analysis found weaker evidence for TGF-β1 869C/T polymorphism and breast cancer risk (Cox et al., 2007), while the other, based on 31 studies, conducted a subgroup analysis to explore the association between 869C/T polymorphisms and HNC risk. This study found that this SNP was associated with increased risk of HNC (TC vs. TT: OR = 1.34, 95%CI: 1.07–1.67; Gu et al., 2015). Conversely, another study showed that the C allele was found to be associated with decreased risk of hepatocellular cancer (Zhang et al., 2012). However, the subgroup analysis in our study found that these overall results may be affected by ethnicity and HWE. Significant association was observed in Asian groups in the allelic model (C vs. T: OR = 1.400, 95%CI: 1.003–1.956, *P* = 0.048) and the homozygote model (CC vs. TT: CC vs. TT: OR = 1.814, 95%CI: 1.018–3.233, *P* = 0.043), while there was no significant association in other populations. After we removed the study derived from HWE, no significant association was found in the overall comparison. Sensitivity analysis performed by sequentially removing one study each time showed that the results of the pooled OR were robust only in the CT vs. TT model, but were not stable in four models: C vs. T, CC vs. TT, CT/CC vs. TT, and CC vs. CT/TT. This instability may be caused by three factors: first, the limited number of the included studies; second, a strong SNP effect with a large 95%CI caused by the small sample size of some included studies; and third, different race and the adjusted confounded factors in the included studies. Therefore, more well-designed studies with larger sample sizes are needed to validate our meta-analysis.

Regarding TGF-β1 915G/C polymorphism, no significant association was observed in all of the comparison models. Only two included studies reported on TGF-β1 915G/C polymorphism and both involved Asian populations, hence no subgroup analysis was conducted. The conclusions of these two studies are also inconsistent. Guar et al. found that 915G/C SNP did not show any significant difference in genotype and allele frequencies between patients and controls (Gaur et al., 2011), while Hsu et al. concluded that the C allele and the GC genotype of TGF-β1 was significantly higher in frequency in cancer patients compared with a healthy control group (Hsu et al., 2015). In a previous study, Niu et al. also found that there was no significant association between 915G/C polymorphisms and the risk of gastric cancer, both in overall analyses and subgroup analyses based on ethnicity (Niu et al., 2012).

In this meta-analysis, the statistical heterogeneity was high in most of the comparison models and the subgroup analyses. Three factors may contribute to the high heterogeneity. First, environmental factors are of importance in the development of cancers, hence well-matched control subjects in a case-control study may produce more reliable results. However, all seven included studies were case-control studies that adjusted different number and kind of confounding factors. This may profoundly affect the accuracy of the results and increase the heterogeneity between the studies. Second, the role of genetic factors may vary in different ethnicities. The findings of the studies on different races may be different, which will increase the heterogeneity between the included studies. Third, the number of studies included in this meta-analysis is limited, especially concerning the TGF-β1−509C/T and 915G/C polymorphism. Moreover, the sample size in the included studies is also limited.

Previously there was a meta-analysis assessing the association in a subgroup analysis, but the authors included only four studies on HNC (Gu et al., 2015). Therefore, to the best of our knowledge, this is the most comprehensive meta-analysis to estimate the association between SNPs in TGF-β1 and HNC risk. In addition, subgroup analyses were performed to explore whether particular characteristics of studies were related to the overall analysis. Nevertheless, there are some limitations that should be addressed. First, one study with low quality or derived from HWE, was included; however, this study was removed when the subgroup analysis was performed. Second, due to the limited number of studies, the publication biases of the included studies have not been analyzed. Third, the studies on−509C/T, 915G/C and the non-Asian population (classified as “others” in this study) are limited, so the related results should be treated with some caution. Fourth, the pooled ORs in the 869T/C were not stable and more studies are needed.

In summary, our meta-analysis indicated that there was no statistical association between TGF-β1−509C/T, 915G/C polymorphism and HNC risk in any genetic models, but we found that TGF-β1 869C/T polymorphism may be involved in the susceptibility to HNC, especially in Asian patients. However, considering the limitations of this study, the results should be interpreted with some caution. More well-designed studies with larger sample sizes and well-matched controls are required to validate our conclusion, especially concerning non-Asian populations.

## Author contributions

HL and NH conceived and designed the experiments; QS and XW performed the literature research, study selection and data extraction; CC and SY performed the section of risk of bias evaluation and data analysis; QS and XW wrote the paper. All authors approved the final version to be published.

### Conflict of interest statement

The authors declare that the research was conducted in the absence of any commercial or financial relationships that could be construed as a potential conflict of interest.

## References

[B1] Al-Hadyan K. S. Al-Harbi N. M. Al-Qahtani S. S. Alsbeih G. A. (2012). Involvement of single-nucleotide polymorphisms in predisposition to head and neck cancer in Saudi Arabia. Genet. Test. Mol. Biomarkers 16, 95–101. 10.1089/gtmb.2011.0126 21877955 PMC3277923

[B2] Bediaga N. G. Marichalar-Mendia X. Rey-Barja N. Setien-Olarra A. Gonzalez-Garcia J. A. De Pancorbo M. M.. (2015). Polymorphisms in alcohol and tobacco metabolism genes in head and neck cancer in the Basque Country. J. Oral Pathol. Med. 44, 769–775. 10.1111/jop.12305 25639971

[B3] Bettendorf O. Piffko J. Bankfalvi A. (2004). Prognostic and predictive factors in oral squamous cell cancer: important tools for planning individual therapy? Oral Oncol. 40, 110–119. 10.1016/j.oraloncology.2003.08.010 14693233

[B4] Bray F. Haugen M. Moger T. A. Tretli S. Aalen O. O. Grotmol T. (2008). Age-incidence curves of nasopharyngeal carcinoma worldwide: bimodality in low-risk populations and aetiologic implications. Cancer Epidemiol. Biomarkers Prev. 17, 2356–2365. 10.1158/1055-9965.EPI-08-0461 18768504

[B5] Brunotto M. Zarate A. M. Bono A. Barra J. L. Berra S. (2014). Risk genes in head and neck cancer: a systematic review and meta-analysis of last 5 years. Oral Oncol. 50, 178–188. 10.1016/j.oraloncology.2013.12.007 24370206

[B6] Cai Q. Tang Y. Zhang M. Shang Z. Li G. Tian J. (2014). *TGF*-β1 Leu10Pro polymorphism contributes to the development of prostate cancer: evidence from a meta-analysis. Tumour Biol. 35, 667–673. 10.1007/s13277-013-1092-5 23975370

[B7] Camargo M. C. Mera R. Correa P. Peek R. M. Jr. Fontham E. T. Goodman K. J.. (2006). Interleukin-1β and interleukin-1 receptor antagonist gene polymorphisms and gastric cancer: a meta-analysis. Cancer Epidemiol. Biomarkers Prev. 15, 1674–1687. 10.1158/1055-9965.EPI-06-0189 16985030

[B8] Carneiro N. K. Oda J. M. Losi Guembarovski R. Ramos G. Oliveira B. V. Cavalli I. J.. (2013). Possible association between TGF-β1 polymorphism and oral cancer. Int. J. Immunogenet. 40, 292–298. 10.1111/iji.12037 23442056

[B9] Chai R. C. Lambie D. Verma M. Punyadeera C. (2015). Current trends in the etiology and diagnosis of HPV-related head and neck cancers. Cancer Med. 4, 596–607. 10.1002/cam4.424 25644715 PMC4402074

[B10] Chang W. W. Zhang L. Su H. Yao Y. S. (2014). An updated meta-analysis of transforming growth factor-β1 gene: three polymorphisms with gastric cancer. Tumour Biol. 35, 2837–2844. 10.1007/s13277-013-1408-5 24254308

[B11] Cox A. Dunning A. M. Garcia-Closas M. Balasubramanian S. Reed M. W. Pooley K. A.. (2007). A common coding variant in CASP8 is associated with breast cancer risk. Nat. Genet. 39, 352–358. 10.1038/ng1981 17293864

[B12] Fan H. Yu H. Deng H. Chen X. (2014). Transforming growth factor-beta1 rs1800470 polymorphism is associated with lung cancer risk: a meta-analysis. Med. Sci. Monit. 20, 2358–2362. 10.12659/MSM.891122 25409890 PMC4247234

[B13] Gaur P. Mittal M. Mohanti B. K. Das S. N. (2011). Functional genetic variants of *TGF*-β1 and risk of tobacco-related oral carcinoma in high-risk Asian Indians. Oral Oncol. 47, 1117–1121. 10.1016/j.oraloncology.2011.07.033 21865076

[B14] Global Burden of Disease Study 2013 Collaborators (2015). Global, regional, and national incidence, prevalence, and years lived with disability for 301 acute and chronic diseases and injuries in 188 countries, 1990-2013: a systematic analysis for the Global Burden of Disease Study 2013. Lancet 386, 743–800. 10.1016/S0140-6736(15)60692-4 26063472 PMC4561509

[B15] Gu Y.-Y. Wang H. Wang S. (2015). TGF-β1 C−509T and T869C polymorphisms and cancer risk: a meta analysis. Int. J. Clin. Exp. Med. 8, 17932–17940. 26770387 PMC4694287

[B16] Hsu H. J. Yang Y. H. Shieh T. Y. Chen C. H. Kao Y. H. Yang C. F.. (2014). Role of cytokine gene (interferon-gamma, transforming growth factor-beta1, tumor necrosis factor-alpha, interleukin-6, and interleukin-10) polymorphisms in the risk of oral precancerous lesions in Taiwanese. Kaohsiung J. Med. Sci. 30, 551–558. 10.1016/j.kjms.2014.09.003 25458044 PMC11916204

[B17] Hsu H. J. Yang Y. H. Shieh T. Y. Chen C. H. Kao Y. H. Yang C. F.. (2015). TGF-beta1 and IL-10 single nucleotide polymorphisms as risk factors for oral cancer in Taiwanese. Kaohsiung J. Med. Sci. 31, 123–129. 10.1016/j.kjms.2014.12.003 25744234 PMC11916862

[B18] Hu S. Zhou G. Zhang L. Jiang H. Xiao M. (2012). The effects of functional polymorphisms in the *TGFβ*1 gene on nasopharyngeal carcinoma susceptibility. Otolaryngol. Head Neck Surg. 146, 579–584. 10.1177/0194599811434890 22282866

[B19] Khaali W. Moumad K. Ben Driss E. K. Benider A. Ben Ayoub W. Hamdi-Cherif M.. (2016). No association between TGF-beta1 polymorphisms and risk of nasopharyngeal carcinoma in a large North African case-control study. BMC Med. Genet. 17:72. 10.1186/s12881-016-0337-8 27733130 PMC5062876

[B20] Lacko M. Braakhuis B. J. Sturgis E. M. Boedeker C. C. Suarez C. Rinaldo A.. (2014). Genetic susceptibility to head and neck squamous cell carcinoma. Int. J. Radiat. Oncol. Biol. Phys. 89, 38–48. 10.1016/j.ijrobp.2013.09.034 24725688

[B21] Li F. Wang J. Chen M. (2016). Single nucleotide polymorphisms in DNA repair genes and the risk of laryngeal cancer: a meta-analysis. Biomed. Pharmacother. 78, 92–100. 10.1016/j.biopha.2015.12.019 26898429

[B22] Li K. Tie H. Hu N. Chen H. Yin X. Peng C.. (2014). Association of two polymorphisms rs2910164 in miRNA-146a and rs3746444 in miRNA-499 with rheumatoid arthritis: a meta-analysis. Hum. Immunol. 75, 602–608. 10.1016/j.humimm.2014.05.002 24824381

[B23] Liu Y. Lin X. F. Lin C. J. Jin S. S. Wu J. M. (2012). Transforming growth factor beta-1 C−509T polymorphism and cancer risk: a meta-analysis of 55 case-control studies. Asian Pac. J. Cancer Prev. 13, 4683–4688. 10.7314/APJCP.2012.13.9.4683 23167402

[B24] Lu S. L. Reh D. Li A. G. Woods J. Corless C. L. Kulesz-Martin M.. (2004). Overexpression of transforming growth factor beta1 in head and neck epithelia results in inflammation, angiogenesis, and epithelial hyperproliferation. Cancer Res. 64, 4405–4410. 10.1158/0008-5472.CAN-04-1032 15231647

[B25] Lu W. Q. Qiu J. L. Huang Z. L. Liu H. Y. (2016). Enhanced circulating transforming growth factor beta 1 is causally associated with an increased risk of hepatocellular carcinoma: a mendelian randomization meta-analysis. Oncotarget 7, 84695–84704. 10.18632/oncotarget.13218 27835897 PMC5356692

[B26] Lundberg M. Leivo I. Saarilahti K. Makitie A. A. Mattila P. S. (2012). Transforming growth factor beta 1 genotype and p16 as prognostic factors in head and neck squamous cell carcinoma. Acta Otolaryngol. 132, 1006–1012. 10.3109/00016489.2012.678944 22667340

[B27] Ma F. Zhang H. Zhai Y. Huang W. Zhao C. Ou S.. (2011). Functional polymorphism−31C/G in the promoter of *BIRC5* gene and risk of nasopharyngeal carcinoma among chinese. PLoS ONE 6:e16748. 10.1371/journal.pone.0016748 21304814 PMC3033414

[B28] Ma L. Zhou N. (2016). Association between an insertion/deletion polymorphism in IL-1A gene and cancer risk: a meta-analysis. Onco. Targets Ther. 9, 1–6. 10.2147/OTT.S95887 26719711 PMC4690651

[B29] Massagué J. (2012). TGFβ signalling in context. Nat. Rev. Mol. Cell Biol. 13, 616–630. 10.1038/nrm3434 22992590 PMC4027049

[B30] Munshi T. Heckman C. J. Darlow S. (2015). Association between tobacco waterpipe smoking and head and neck conditions: a systematic review. J. Am. Dent. Assoc. 146, 760–766. 10.1016/j.adaj.2015.04.014 26409986

[B31] Nachman M. W. (2001). Single nucleotide polymorphisms and recombination rate in humans. Trends Genet. 17, 481–485. 10.1016/S0168-9525(01)02409-X 11525814

[B32] Niu H. Niu Z. Zhang X. L. Chen Z. L. (2012). Absence of association between transforming growth factor B1 polymorphisms and gastric cancer: a meta-analysis. DNA Cell Biol. 31, 706–712. 10.1089/dna.2011.1426 22074128

[B33] Petersen P. E. (2009). Oral cancer prevention and control–the approach of the World Health Organization. Oral Oncol. 45, 454–460. 10.1016/j.oraloncology.2008.05.023 18804412

[B34] Rich J. Borton A. Wang X. (2001). Transforming growth factor-β signaling in cancer. Microsc. Res. Technol. 52, 363–373. 10.1002/1097-0029(20010215)52:4<363::AID-JEMT1021>3.0.CO;2-F 11170295

[B35] Rosenthal E. Mccrory A. Talbert M. Young G. Murphy-Ullrich J. Gladson C. (2004). Elevated expression of TGF-β1 in head and neck cancer-associated fibroblasts. Mol. Carcinog. 40, 116–121. 10.1002/mc.20024 15170816

[B36] Shukla P. Gupta D. Pant M. C. Parmar D. (2012). CYP 2D6 polymorphism: a predictor of susceptibility and response to chemoradiotherapy in head and neck cancer. J. Cancer Res. Ther. 8, 40–45. 10.4103/0973-1482.95172 22531512

[B37] Siegel P. M. Massagué J. (2003). Cytostatic and apoptotic actions of TGF-beta in homeostasis and cancer. Nat. Rev. Cancer 3, 807–821. 10.1038/nrc1208 14557817

[B38] Singh P. K. Bogra J. Chandra G. Ahmad M. K. Gupta R. Kumar V.. (2015). Association of TNF-α (-238 and -308) promoter polymorphisms with susceptibility of oral squamous cell carcinoma in North Indian population. Cancer Biomark. 15, 125–131. 10.3233/CBM-140444 25519014 PMC12928520

[B39] Sivadas V. P. George N. A. Kattoor J. Kannan S. (2013). Novel mutations and expression alterations in SMAD3/TGFBR2 genes in oral carcinoma correlate with poor prognosis. Genes Chromosomes Cancer 52, 1042–1052. 10.1002/gcc.22099 23913824

[B40] Tang B. Vu M. Booker T. Santner S. J. Miller F. R. Anver M. R.. (2003). TGF-beta switches from tumor suppressor to prometastatic factor in a model of breast cancer progression. J. Clin. Invest. 112, 1116–1124. 10.1172/JCI200318899 14523048 PMC198530

[B41] Wei Y. S. Zhu Y. H. Du B. Yang Z. H. Liang W. B. Lv M. L.. (2007). Association of transforming growth factor-beta1 gene polymorphisms with genetic susceptibility to nasopharyngeal carcinoma. Clin. Chim. Acta 380, 165–169. 10.1016/j.cca.2007.02.008 17368597

[B42] Zafereo M. E. Sturgis E. M. Aleem S. Chaung K. Wei Q. Li G. (2009). Glutathione S-transferase polymorphisms and risk of second primary malignancy after index squamous cell carcinoma of the head and neck. Cancer Prev. Res. 2, 432–439. 10.1158/1940-6207.CAPR-08-0222 19401526 PMC2698715

[B43] Zhang C. F. Wang Z. W. Hou M. X. Li K. Zhou X. Xia Y. H. (2012). Transforming growth factor beta1−509C/T and +869T/C polymorphisms on the risk of upper digestive tract cancer: a meta-analysis based on 10,917 participants. Ann. Hum. Genet. 76, 363–376. 10.1111/j.1469-1809.2012.00717.x 22724518

[B44] Zou Y. Song T. Yu W. Zhao R. Wang Y. Xie R.. (2014). XRCC3 polymorphisms are associated with the risk of developing radiation-induced late xerostomia in nasopharyngeal carcinoma patients treated with intensity modulation radiated therapy. Jpn. J. Clin. Oncol. 44, 241–248. 10.1093/jjco/hyt202 24453273

