# Supplementary material for: Association between TGF-β1 Polymorphisms and Head and Neck Cancer Risk: A Meta-Analysis
Source: Front Genet. 2017 Nov 3;8:169. doi: 10.3389/fgene.2017.00169 (PMC5675865; doi:10.3389/fgene.2017.00169)
Supplement: Supplementary file 1 [file Table1.DOCX]

Supplementary Material

**Association between TGF-β1 Polymorphisms and Head and Neck Cancer Risk: A Meta-Analysis**

**Quan Shi ^1#^, Xing Wang ^2#^, Chuan Cai ^1^, Shuo Yang ^1^, Na Huo ^1*^, Hongchen Liu ^1*^**

^1^ Institute of Stomatology, Chinese PLA General Hospital, Beijing, China

^2^ Shanxi medical university stomatological hospital, Taiyuan, China

*** Correspondence:**Na Huo
huona301@sina.cn

Hongchen Liu
liuhc301@hotmail.com

^#^ These authors contributed equally to this work.

| **Table S1. Scale for methodological quality assessment.** | |
| --- | --- |
| Criteria | Score |
| 1.Representativeness of cases |  |
| Cancers diagnosed according to acknowledged criteria. | 2 |
| Mentioned the diagnosed criteria but not specifically described. | 1 |
| Not Mentioned. | 0 |
| 2.Source of controls |  |
| Population or community based | 3 |
| Hospital-based cancer-free controls | 2 |
| Healthy volunteers without total description | 1 |
| Cancer-free controls with related diseases | 0.5 |
| Not described | 0 |
| 3.Sample size |  |
| >200 | 2 |
| 100-200 | 1 |
| <100 | 0 |
| 4.Quality control of genotyping methods |  |
| Repetition of partial/total tested samples with a different method | 2 |
| Repetition of partial/total tested samples with the same method | 1 |
| Not described | 0 |
| 5.Hardy-Weinberg equilibrium (HWE) |  |
| Hardy-Weinberg equilibrium in control subjects | 1 |
| Hardy-Weinberg disequilibrium in control subjects | 0 |
