# Supplementary material for: Association between TGF-β1 Polymorphisms and Head and Neck Cancer Risk: A Meta-Analysis
Source: Front Genet. 2017 Nov 3;8:169. doi: 10.3389/fgene.2017.00169 (PMC5675865; doi:10.3389/fgene.2017.00169)
Supplement: Supplementary file 2 [file Table2.DOCX]

Supplementary Material

**Association between TGF-β1 Polymorphisms and Head and Neck Cancer Risk: A Meta-Analysis**

**Quan Shi ^1#^, Xing Wang ^2#^, Chuan Cai ^1^, Shuo Yang ^1^, Na Huo ^1*^, Hongchen Liu ^1*^**

^1^ Institute of Stomatology, Chinese PLA General Hospital, Beijing, China

^2^ Shanxi medical university stomatological hospital, Taiyuan, China

*** Correspondence:**Na Huo
huona301@sina.cn

Hongchen Liu
liuhc301@hotmail.com

^#^ These authors contributed equally to this work.

**Table S2. Sensitivity analysis performed by sequential removing one study for the 869T/C.**

| **Model** | **Study exclude** | **OR** | **95% CI** | **P** |
| --- | --- | --- | --- | --- |
| **C vs T** | **Total** | **1.351** | **1.030-1.772** | **0.030** |
|  | Khaali W 2016 | 1.503 | 1.077-2.089 | 0.017 |
|  | Carneiro NK 2013 | 1.273 | 0.976-1.660 | 0.075 |
|  | Hu S 2012 | 1.488 | 1.026-2.156 | 0..036 |
|  | Al-Hadyan KS 2012 | 1.435 | 1.023-2.013 | 0.036 |
|  | Gaur P 2011 | 1.187 | 0.946-1.489 | 0.138 |
|  | Wei YS 2007 | 1.290 | 0.965-1.725 | 0.086 |
| **CC vs TT** | **Total** | **1.585** | **1.026-2.449** | **0.038** |
|  | Khaali W 2016 | 1.865 | 1.125-3.091 | 0.016 |
|  | Carneiro NK 2013 | 1.514 | 0.947-2.419 | 0.083 |
|  | Hu S 2012 | 1.818 | 1.016-3.253 | 0.044 |
|  | Al-Hadyan KS 2012 | 1.701 | 0.991-2.921 | 0.054 |
|  | Gaur P 2011 | 1.312 | 0.910-1.892 | 0.146 |
|  | Wei YS 2007 | 1.429 | 0.918-2.225 | 0.113 |
| **CT vs TT** | **Total** | **1.279** | **0.957-1.709** | **0.097** |
|  | Khaali W 2016 | 1.408 | 0.969-2.045 | 0.073 |
|  | Carneiro NK 2013 | 1.163 | 0.912-1.484 | 0.223 |
|  | Hu S 2012 | 1.396 | 0.933-2.089 | 0.104 |
|  | Al-Hadyan KS 2012 | 1.395 | 0.994-1.959 | 0.054 |
|  | Gaur P 2011 | 1.146 | 0.878-1.495 | 0.315 |
|  | Wei YS 2007 | 1.265 | 0.914-1.750 | 0.157 |
| **CT/CC vs TT** | **Total** | **1.389** | **1.008-1.937** | **0.044** |
|  | Khaali W 2016 | 1.568 | 1.051-2.340 | 0.028 |
|  | Carneiro NK 2013 | 1.290 | 0.938-1.774 | 0.117 |
|  | Hu S 2012 | 1.545 | 0.991-2.406 | 0.055 |
|  | Al-Hadyan KS 2012 | 1.526 | 1.026-2.269 | 0.037 |
|  | Gaur P 2011 | 1.198 | 0.912-1.574 | 0.195 |
|  | Wei YS 2007 | 1.338 | 0.942-1.900 | 0.104 |
| **CC vs CT/TT** | **Total** | **1.314** | **0.953-1.810** | **0.095** |
|  | Khaali W 2016 | 0.862 | 0.589-1.261 | 0.444 |
|  | Carneiro NK 2013 | 1.155 | 0.549-2.432 | 0.704 |
|  | Hu S 2012 | 1.017 | 0.781-1.324 | 0.899 |
|  | Al-Hadyan KS 2012 | 1.362 | 0.841-2.206 | 0.209 |
|  | Gaur P 2011 | 2.818 | 1.348-5.891 | 0.006 |
|  | Wei YS 2007 | 2.076 | 1.176-3.665 | 0.012 |

OR: Odds ratio; CI: Confidence interval; P: P values for pooled ORs.
